# A New Multiplex Assay of 17 Autosomal STRs and Amelogenin for Forensic Application

**DOI:** 10.1371/journal.pone.0057471

**Published:** 2013-02-25

**Authors:** Suhua Zhang, Huaizhou Tian, Jun Wu, Shumin Zhao, Chengtao Li

**Affiliations:** 1 Shanghai Key Laboratory of Forensic Medicine, Institute of Forensic Sciences, Ministry of Justice, Shanghai, People’s Republic of China; 2 PEOPLESPOTINC Research & Development, Beijing, People’s Republic of China; MOE Key Laboratory of Environment and Health, School of Public Health, Tongji Medical College, Huazhong University of Science and Technology, China

## Abstract

This paper describes a newly devised autosomal short tandem repeat (STR) multiplex polymerase chain reaction (PCR) systems for 17 autosomal loci (D1S1656, D2S441, D3S1358, D3S3045, D6S477, D7S3048, D8S1132, D10S1435, D10S1248, D11S2368, D13S325, D14S608, D15S659, D17S1290, D18S535, D19S253 and D22-GATA198B05) and Amelogenin. Primers for the loci were designed and optimized so that all of the amplicons were distributed from 50 base pairs (bp) to less than 500 bp within a five-dye chemistry design with the fifth dye reserved for the sizing standard. Strategies were developed to overcome challenges that encountered in creating the final assay. The limits of the multiplex were tested, resulting in the successful amplification of genomic DNA range from 0.25–4 ng with 30 PCR cycles. A total of 681 individuals from the Chinese Han population were studied and forensic genetic data were present. No significant deviations from Hardy–Weinberg equilibrium were observed. A total of 180 alleles were detected for the 17 autosomal STRs. The cumulative mean exclusion chance in duos (CMEC_D_) was 0.999967, and cumulative mean exclusion chance in trios (CMEC_T_) was 0.99999995. We conclude that the present 17plex autosomal STRs assay provides an additional powerful tool for forensic applications.

## Introduction

The study of genetic variation, using DNA polymorphisms distributed throughout the genome, has allowed better understanding of the history and diversity of human populations as well as providing a system for forensic testing [1], [2]. At present, STR typing can be considered a standard approach and the method of choice in the forensic field, allowing a high discrimination power adequate for addressing most problems of human identification and paternity testing [3]. Analysis of STR loci is an essential component of forensic genetics. 13 core STR loci of Combined DNA Index System (CODIS) which used for a DNA database of felon were widely used in commercial kits [4]. On the other hand, many highly polymorphic STR loci exist, which are unlinked to the core STR loci (non-CODIS loci). Analyses of such polymorphic non-CODIS loci in addition to the core CODIS loci may increase the power of exclusion in complex kinship testing [4], [5].

The present study sought to develop a new multiplex PCR system for simultaneous typing of highly polymorphic non-CODIS loci. We selected 16 polymorphic non-CODIS STR loci (D1S1656, D2S441, D3S3045, D6S477, D7S3048, D8S1132, D10S1435, D10S1248, D11S2368, D13S325, D14S608, D15S659, D17S1290, D18S535, D19S253 and D22-GATA198B05) and a CODIS STR locus (D3S1358) for total 17 STR loci. This paper described the new development of the 17plex autosomal STRs assay and surveyed their efficacy for forensic application.

## Materials and Methods

### Ethics Statement

Human blood samples were collected with the approval of the Ethics Committee of the Institute of Forensic Sciences, Ministry of Justice, P.R. China. Samples were obtained from unrelated volunteers after receiving written informed consents. Animal blood samples were collected with the approval of the Animal Use Committee of the Institute of Forensic Sciences, Ministry of Justice, P. R. China.

### (a) Candidate STRs Selection

The initial candidate pool of autosomal STRs for this study was based on the STR DNA Internet DataBase created by John M. Butler et al (http://www.cstl.nist.gov/strbase/) and National Center for Biotechnology Information (NCBI) (http://www.ncbi.nlm.nih.gov/).

STRs were then selected according to the following criteria: (I) non-coding, non-CODIS, autosomal STRs; (II) average heterozygosity ≥0.30 in European, African and Asian population groups; (III) located on different chromosomes, if not, at most two STRs were selected from the same chromosome and make sure on different arms; and (IV) physical distance between the selected STRs and CODIS STRs above 10 Mb.

After applying the described criteria, 16 ideal candidate STRs were obtained. Flanking sequences of these STRs, as well as reported sequence variants within these region, were obtained using the University of California Santa Cruz Genome Browser (Human February 2009 Assembly; GRCh37/hg19) at http://genome.ucsc.edu/.

Locus D3S1358 which belonged to CODIS STRs was added to our final assay, for comparing the accuracy of the final multiplex and usually used commercial kit (e.g. AmpFℓSTR® Identifiler® Kit). The sex-typing locus Amelogenin was also included in the final assay.

### (b) Primer and Multiplex Assay Design

PCR primer designing was performed using software of primer premier v5.0 and Oligo v6.0, applying the following main criteria: primer length of 18–30 bp; amplicon length of 50–500 bp; optimum Tm from 56°C to 64°C; optimum CG content range from 45% to 55%. Obtained primer pairs were then checked for non-specific hybridizations in other genome regions using NCBI Basic Local Alignment Search Tool (BLAST) at http://blast.ncbi.nlm.nih.gov/. AutoDimer software was also applied to screen candidate primer sequences for inter-primers compatibility.

Forward primer of each STR was labelled at the 5′ end with either 6 FAM™, HEX™, TEMRA™ and ROX™ (Applied Biosystems, Foster City, CA). All primers were synthesized and labelled by Sangon Biotech. Co. Ltd (Shanghai, China).

All selected STRs were then organized by expected amplicon length and assigned into four different dye-labelling fluorochromes in order to achieve an evenly balanced genotyping assay for a single PCR and electrophoretic separation.

### (c) PCR Amplification and STRs Detection

All selected STRs were initially amplified in singleplex, in order to evaluate primer performance and expected allele sizes. After optimization, the amplification of all STRs was performed in a single multiplex PCR including 1×PCR buffer, 200 µM dNTP, 1.5 mM MgCl_2_, 1 U GoldTaq DNA polymerase, 5 µL primer mix and 0.25–4 ng of genomic DNA in a 25 µL final reaction volume. Thermocycling condition for the multiplex PCR assay was initial incubation at 95°C for 11 min; 30 cycles at 94°C for 30 s, 60°C for 60 s and 70°C for 60 s; with a final extension at 60°C for 30 min.

PCR products were subsequently prepared for capillary electrophoresis by adding 1 µL of each amplified product to 9 µL of a 17∶1 mixture of Hi-Di formamide and Orange-500 size standard, respectively. Separation and detection were performed with AB 3130*xl* Genetic Analyzer (Applied Biosystems, Foster City, CA) using filter set E5 and POP4 polymer (Applied Biosystems, Foster City, CA). Samples were injected electrokinetically for 5 sec at 3 kV. The STR alleles were then separated at 15 kV at a run temperature of 60°C. Genotyping data of studied samples were then collected with GeneMapper v3.2.1 software (Applied Biosystems, Foster City, CA). Positive control DNA of 9947A human cell line samples (Applied Biosystems, Foster City, CA) was used to test the overall performance of the multiplex genotyping assay.

### (d) Sensitivity Testing

To determine the minimum quantity of DNA required to achieve reliable results (peak highs at all loci exceeded 100 relative fluorescence units (RFU)) with the multiplex assay, we used aliquots of the cell line 9947A DNA (50, 100, 250, 500 pg, 1, 2, 4 and 5 ng). Every concentration tested for three replicates.

### (e) Specificity Testing

To determine the specificity of this multiplex assay, several kinds of living creatures’ bloods (Human, Pig, Chick, Duck, Dog and Cat) were tested.

### (f) Population Samples for STR Polymorphism Analysis

Peripheral bloods were collected from a total of 681 unrelated healthy individuals (343 males and 338 females) of Chinese Han population, the largest nation of China. All samples of the volunteers were genotyped with commercial kit of AmpF*ℓ*STR® Identifiler® PCR Amplification Kit before.

Genomic DNA was extracted using the Chelex-100 protocol as described by Walsh et al [6]. The quantity of recovered DNA was determined by spectrophotometric method.

### (g) Statistical Analysis

Hardy-Weinberg equilibrium and allele frequencies of the 17 autosomal STR were calculated by GeneAlEx 6.5 software (Genetic analysis in Excel) [7]. Statistical parameters to evaluate the forensic efficiency, such as power of discrimination (DP), heterozygosity (HET), mean exclusion chance in duos (MEC_D_), mean exclusion chance in trios (MEC_T_),polymorphism information content (PIC), and Genediversity (GD) were assessed by PowerMarker V3.25 and Cervus V2.0 software [1], [8].

## Results and Discussion

### (a) Primer and Multiplex PCR Optimization

The primer sequences, T_m_ values and concentrations of the 17 autosomal STR loci and Amelogenin locus in the final multiplex assay were listed in Table 1. Primers for each STR locus were initially tested in singleplex to evaluate the performance. The criteria for primer “failure” in this study is defined as those that produce profiles that exhibit incomplete adenylation, the presence of PCR artifacts, nonspecific products, low signal, or no PCR product at all [5]. With those “failure” ones, singleplex PCR optimization were adopted, otherwise, re-designing the PCR primers. Once the successful primers at locus were determined, those were combined together and tested in multiplex. At first, the tested multiplex assay indicated a high degree of baseline noises and many profiles displayed the features of failure. This was due to multiplex primer incompatibility. Based on the data of primer concentrations and peak heights, optimizations of the multiplex assay were performed.

**Table 1:** Primer information of STR loci included in the new studied kit.

STR locus	Dye Label	Primer Sequence:(5′–3′)	Primer Concentration(µM)	Tm	fragment size range
D6S477	6-FAM™	F: GGCTGATGAGGTGAAATATTTGC	0.14	60.7	100–165
		R: GATATCTCAAACAACCTCAACAACA	0.14	58.2	
D22-GATA198B05	6-FAM™	F: AGCCAGCATTCTTAAAACTCTAAGG	0.2	60.5	170–240
		R: ATCAGCCCTGTGACAGAAGTAAATA	0.2	59.5	
D15S659	6-FAM™	F: TGATTTGCCATGATAGATGGCT	0.16	59.8	245–330
		R: GTTTGGGACTTCAAAGACAAAAGA	0.16	59.8	
D8S1132	6-FAM™	F: ATCACATCCTTGTTTCCTCATTTTATT	0.5	59.4	335–450
		R: AGATTGCGTCACTGCACTCC	0.5	58.2	
Amelogenin	HEX™	F: CCCTGGGCTCTGTAAAGAATAG	0.2	58	102–108
		R: ATCAGAGCTTAAACTGGGAAGCTG	0.2	61.5	
D3S1358	HEX™	F: TATGATTCCCCCACTGCAGT	0.1	57.5	115–160
		R: ATGAAATCAACAGAGGCTTGC	0.1	57.1	
D3S3045	HEX™	F: TAGATGGCTTCCTAGGCTCAATTAG	0.24	61	186–255
		R: ATAAATATTTCTGTTTCTCCTGGGG	0.24	59.9	
D17S1290	HEX™	F: TTCAGTTAGCCAAGATAATGCCA	0.26	59.7	265–380
		R: GTAAGGCTGAGTTCCTCTGGGT	0.26	59.3	
D14S608	HEX™	F: TACTACCTCTTCAGTGAGCTTTCGT	0.5	59.2	388–450
		R: CTGGCAACAATGATTCTATTTTCTC	0.5	59.7	
D2S441	TEMRA™	F: GAACTGTGGCTCATCTATGAAAACT	0.15	59.1	80–122
		R: GCTAAGTGGCTGTGGTGTTATGATA	0.15	60.7	
D18S535	TEMRA™	F: CACACCCATAACTTTTTTCCTCTAG	0.25	59.1	130–170
		R: GCAGGCTAAGAGTTACCCATAATTA	0.25	59.1	
D13S325	TEMRA™	F: TATCTCCAGAATCCTTCCAGCTAAT	0.15	60.3	175–265
		R: CTGGAATAACTATTCATGCTAACCA	0.15	58.1	
D10S1435	TEMRA™	F: TGCATTGAGTTTTATTCTGTTTATC	0.5	56.2	275–350
		R: AGTACCATGTGAATCTTCATCTTCC	0.5	58.7	
D11S2368	TEMRA™	F: GCATTGGCTGTCATAATATTCACTC	0.5	60.2	355–420
		R: TATACATCTCCTCCAAGAGCTTTCC	0.5	60.6	
D1S1656	ROX™	F: GTGTTGCTCAAGGGTCAACTGTATG	0.2	62.8	115–170
		R: AGAGAAATAGAATCACTAGGGAACC	0.2	57.1	
D7S3048	ROX™	F: GCATTGCCACTTCCCCCTG	0.5	63.1	175–233
		R: CCCCCGCAGTCAAAAATCT	0.5	59.8	
D10S1248	ROX™	F: GAGCATTAGCCCCAGGACCA	0.5	62.2	234–280
		R: TGCAGTGCTTGGCAAAGAGC	0.5	62.2	
D19S253	ROX™	F: CTCCATGAACAGACAGTTTGTCTTC	0.5	59.9	355–420
		R: CTTTTCAGCTTCCATAAACATGAGT	0.5	59.4	

**Figure 1:**
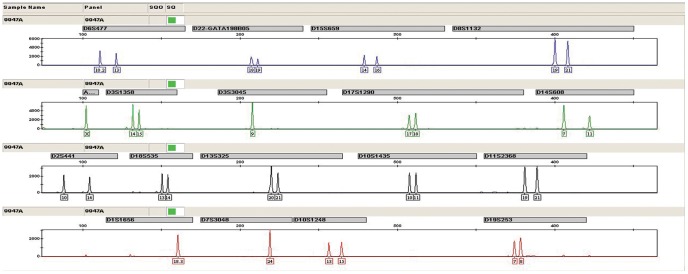
The genotyping profile of positive control DNA of 9947A with the 17plex assay.

**Figure 2:**
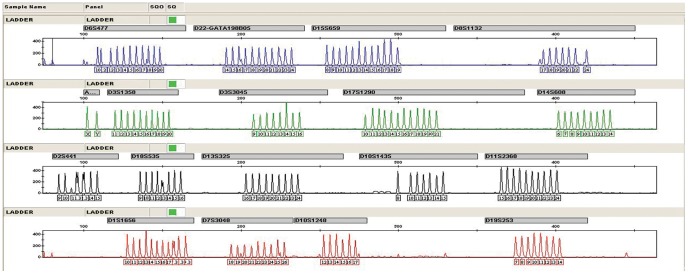
The genotyping profile of ladder with the 17plex assay.

The final assay design resulted in a good working base to set up the multiplex reaction. In the initial multiplex tests some primer pairs revealed weaker performance than others, as would be expected since the optimum annealing temperature was not exactly the same for all primers. To minimize these effects and aiming for a more balanced result, we tested different primer mix concentrations and annealing temperatures. After the multiplex development and optimization, 17 autosomal STRs plus Amelogenin were successfully amplified in a single PCR reaction, following the final optimum conditions. The genotyping profiles of positive control DNA of 9947A and ladder were showed in Figure 1 and Figure 2, respectively.

### (b) Multiplex PCR Performance

In general, during the genotyping of 681 unrelated population samples, the multiplex reaction revealed to be robust and able to successfully amplify all STRs in samples with 0.25–4 ng DNA in a 25 µL final reaction volume. The genotyping results of locus D3S1358 by our new multiplex assay and the commercial kit of AmpFℓSTR® Identifiler® Kit were identical with each other.

Several DNA concentrations ranging from 50 pg to 5 ng were tested with the multiplex, using dilution series of positive control sample 9947A. The best results, revealing an improved peak balance among STRs, were observed when using 0.5–1 ng of DNA in a 25 µL PCR final volume, although good results for all STRs were obtained for concentrations from 0.25 to 4 ng. Nevertheless, when using highly concentrated DNA samples (>4 ng), we observed strong signals and over-scaled peaks; in these cases, the presence of pull-ups and abnormally shaped peaks due to ineffective fluorescence correction can make interpretation of the genotyping results difficult. Furthermore, the use of higher amounts of DNA in the reaction occasionally caused the inhibition of amplification of longer size amplicons. In samples with DNA amounts as low as 0.1–0.25 ng, it was still possible to amplify all markers; nonetheless, the results were not always consistent, sometimes presenting unusual allele imbalances or allele dropouts observed from the known consensus genotypes of the control DNAs used. The marked heterozygote imbalance or allele dropouts with small quantities of target DNA have been previously reported and are a consequence of stochastic variation in PCR amplification when the number of template molecules is very low [9]. We highlight the fact that, in low copy number applications of this multiplex, the results must be interpreted with caution, and duplicate analyses are required to minimize errors.

As a practical application for forensic use, the multiplex assay was employed in several kinds of living creatures’ bloods (Human, Pig, Chick, Duck, Dog, Cat), aiming to confirm the specificity. Only human blood can have positive results. There are no detected signals for other creatures at any of these 18 STRs included in the kit, which proved that this kit is only suitable for human testing.

### (c) LD Information of 17 Autosomal STRs

Detail information of 17 autosomal STRs (16 non-CODIS STR and a CODIS STR of D3S1358) in the multiplex was listed in Table S1. According to the STRs selection criteria, 14 STRs (D1S1656, D2S441, D3S3045, D6S477, D7S3048, D8S1132, D11S2368, D13S325, D14S608, D15S659, D17S1290, D18S535, D19S253 and D22GATA198B05) located on discrete chromosomes were selected. Although D10S1435 and D10S1248 are found on the same chromosomes (chromosome 10), they are located on different arms (10p15.3 and 10q26.5, respectively) and separated by a considerable distance. In addition, the physical distances between the 16 selected non-CODIS STRs and 13 CODIS STRs are more than 10 Mb.

Genetic linkage disequilibrium (LD) in the human genome would not for consideration if the physical distances are more than 10 Mb [10], [11], [12]. Thus, LD between the current 16 non-CODIS STR and the CODIS core loci may be excluded from consideration. Analysis of extended STR loci using both the present multiplex system and commercial kits which include the CODIS core loci have been demonstrated to be remarkably effective in cases involving difficult kinship testing.

### (d) Forensic Efficiency in Han Populations

The polymorphism analysis of 17 autosomal STRs was studied in the Han population of China. All studied STRs followed Hardy–Weinberg equilibrium. In Table 2 we present allele frequencies derived from the population and the forensic efficiency values for each STR. A total of 180 alleles were detected for the 17 autosomal STR loci while locus D1S1656 had the greatest number of variants, with 15 alleles. All loci show high forensic efficiency with DP values above 0.8. The mean HET value of the STR set was 0.7934, and all loci showed HET values higher than 0.7 except locus D2S411. The highest PIC was 0.8571, at locus D7S3048, while the lowest PIC was 0.6747, at D3S1358. And GD values range from 0.7219 (D3S1358) to 0.8709 (D7S3048).

**Table 2:** Frequencies and forensic parameters of 17 autosomal STRs.

Allele	D1S1656	D2S441	D3S1358	D3S3045	D6S477	D7S3048	D8S1132	D10S1435	D10S1248	D11S2368	D13S325	D14S608	D15S659	D17S1290	D18S535	D19S253	D22GATA198B05
6												0.0646					
7												0.1931				0.1696	
8								0.0308	0.0007			0.0250	0.0051		0.0308	0.0345	
9		0.0007		0.3774								0.1322	0.0015		0.1858	0.0022	
9.3		0.0022															
10	0.0073	0.2504		0.0228	0.0103			0.0433	0.0007			0.2394	0.0059	0.0411	0.0419	0.0206	
10.3		0.0132															
11	0.0609	0.3414	0.0007	0.0308	0.0051			0.1557	0.0066			0.1909	0.1571	0.0477	0.0184	0.1586	
11.2					0.0015												
11.3		0.0455															
12	0.0617	0.1711	0.0007	0.1358	0.0690			0.3789	0.0756			0.1131	0.2173	0.0044	0.0808	0.3311	
12.2															0.0110		
12.3		0.0066															
13	0.1050	0.0316	0.0073	0.2070	0.2078			0.2386	0.3796			0.0367	0.1043	0.0103	0.1997	0.2070	
13.2															0.0007		
13.3		0.0029															
14	0.0786	0.1138	0.0573	0.1681	0.1931			0.1322	0.2093			0.0051	0.0360	0.0198	0.2915	0.0624	0.0066
14.3	0.0044																
15	0.2812	0.0184	0.3392	0.0529	0.3025			0.0176	0.2137	0.0022			0.1762	0.2115	0.1322	0.0140	0.0184
15.3	0.0132																
16	0.2107	0.0022	0.3561	0.0051	0.1615	0.0022	0.0140	0.0029	0.0969	0.0330	0.0044		0.1667	0.3040	0.0073		0.0925
16.3	0.0125																
17	0.0727		0.1696		0.0374	0.0044	0.1028		0.0169	0.1329	0.0044		0.1035	0.1689			0.1557
17.3	0.0470																
18	0.0132		0.0646		0.0073	0.0932	0.2070			0.1182	0.0389		0.0242	0.1167			0.0675
18.3	0.0286																
19			0.0029		0.0044	0.0712	0.2137			0.1791	0.2548		0.0022	0.0565			0.0778
19.3	0.0029																
20			0.0015			0.1872	0.1549			0.1960	0.2651			0.0117			0.0991
21						0.1204	0.1424			0.2093	0.2129			0.0073			0.2930
22						0.0903	0.1021			0.0756	0.1468						0.1659
23						0.1571	0.0485			0.0433	0.0448						0.0184
24						0.1564	0.0147			0.0103	0.0213						0.0051
25						0.0881					0.0051						
26						0.0257											
27						0.0037					0.0015						
**DP**	0.9591	0.9172	0.8754	0.9116	0.9288	0.9696	0.9561	0.9044	0.9009	0.9571	0.9267	0.9504	0.9572	0.9415	0.9407	0.9253	0.9529
**HET**	0.8238	0.6990	0.7327	0.7592	0.7518	0.8840	0.8399	0.7239	0.7504	0.8385	0.8135	0.8399	0.8385	0.7974	0.7827	0.7827	0.8311
**MEC_D_**	0.5325	0.3937	0.3097	0.3770	0.4227	0.5829	0.5174	0.3615	0.3533	0.5220	0.4181	0.4947	0.5222	0.4641	0.4607	0.4128	0.5040
**MEC_T_**	0.6971	0.5722	0.4825	0.5574	0.6005	0.7387	0.6856	0.5411	0.5326	0.6893	0.5954	0.6659	0.6896	0.6385	0.6356	0.5915	0.6742
**PIC**	0.8267	0.7428	0.6747	0.7311	0.7661	0.8571	0.8241	0.7193	0.7139	0.8263	0.7631	0.8110	0.8267	0.7894	0.7890	0.7582	0.8136
**GD**	0.8429	0.7749	0.7219	0.7637	0.7956	0.8709	0.8435	0.7547	0.7510	0.8453	0.7939	0.8326	0.8457	0.8129	0.8134	0.7879	0.8327

Since all of the 17 autosomal STR loci were independent from each other, the combined forensic efficiency parameters were calculated based on allele frequencies while cumulative mean exclusion chance in duos (CMEC_D_) was 0.999967, and cumulative mean exclusion chance in trios (CMEC_T_) was 0.99999995. These results suggest that the 17 autosomal STR loci included in the multiplex assay are highly polymorphic and informative in the Han population of China.

### Concluding Remarks

In this study, we displayed a simple and sensitive 17plex STRs new assay for forensic application, which allows the simultaneous genotyping of 17 autosomal STRs plus Amelogenin presenting high polymorphic in Chinese Han population. The development of this 17plex assay provides support for the use of additional loci for complex kinship testing involving deficient paternity cases and for use in missing person/mass disaster cases.

## Supporting Information

Table S1 **General information for the 17 autosomal STRs included in the final multiplex.**
(DOCX)
