# Correction: A New Multiplex Assay of 17 Autosomal STRs and Amelogenin for Forensic Application

**DOI:** 10.1371/journal.pone.0217892

**Published:** 2019-06-05

**Authors:** Suhua Zhang, Huaizhou Tian, Jun Wu, Shumin Zhao, Chengtao Li

There are errors in Table 1. The primer sequences and Tm values of D15S659 in Table 1 were incorrectly listed. Please see the correct Table 1 here.

**Table 1:** Primer information of STR loci included in the new studied kit.

STR locus	Dye Label	Primer Sequence: (5’-3’)	Primer Concentration (μM)	Tm	fragment size range
D6S477	6-FAM^™^	F: GGCTGATGAGGTGAAATATTTGC	0.14	60.7	100–165
		R: GATATCTCAAACAACCTCAACAACA	0.14	58.2	
D22-GATA198B05	6-FAM^™^	F: AGCCAGCATTCTTAAAACTCTAAGG	0.2	60.5	170–240
		R: ATCAGCCCTGTGACAGAAGTAAATA	0.2	59.5	
D15S659	6-FAM^™^	F: TTTCCCTTCTATTGTGGTAGTCTGG	0.16	60.05	245–330
		R: GAAACTTTGAGTATTTTATGTGTGGAT	0.16	56.87	
D8S1132	6-FAM^™^	F: ATCACATCCTTGTTTCCTCATTTTATT	0.5	59.4	335–450
		R: AGATTGCGTCACTGCACTCC	0.5	58.2	
Amelogenin	HEX^™^	F: CCCTGGGCTCTGTAAAGAATAG	0.2	58	102–108
		R: ATCAGAGCTTAAACTGGGAAGCTG	0.2	61.5	
D3S1358	HEX^™^	F: TATGATTCCCCCACTGCAGT	0.1	57.5	115–160
		R: ATGAAATCAACAGAGGCTTGC	0.1	57.1	
D3S3045	HEX^™^	F: TAGATGGCTTCCTAGGCTCAATTAG	0.24	61	186–255
		R: ATAAATATTTCTGTTTCTCCTGGGG	0.24	59.9	
D17S1290	HEX^™^	F: TTCAGTTAGCCAAGATAATGCCA	0.26	59.7	265–380
		R: GTAAGGCTGAGTTCCTCTGGGT	0.26	59.3	
D14S608	HEX^™^	F: TACTACCTCTTCAGTGAGCTTTCGT	0.5	59.2	388–450
		R: CTGGCAACAATGATTCTATTTTCTC	0.5	59.7	
D2S441	TEMRA^™^	F: GAACTGTGGCTCATCTATGAAAACT	0.15	59.1	80–122
		R: GCTAAGTGGCTGTGGTGTTATGATA	0.15	60.7	
D18S535	TEMRA^™^	F: CACACCCATAACTTTTTTCCTCTAG	0.25	59.1	130–170
		R: GCAGGCTAAGAGTTACCCATAATTA	0.25	59.1	
D13S325	TEMRA^™^	F: TATCTCCAGAATCCTTCCAGCTAAT	0.15	60.3	175–265
		R: CTGGAATAACTATTCATGCTAACCA	0.15	58.1	
D10S1435	TEMRA^™^	F: TGCATTGAGTTTTATTCTGTTTATC	0.5	56.2	275–350
		R: AGTACCATGTGAATCTTCATCTTCC	0.5	58.7	
D11S2368	TEMRA^™^	F: GCATTGGCTGTCATAATATTCACTC	0.5	60.2	355–420
		R: TATACATCTCCTCCAAGAGCTTTCC	0.5	60.6	
D1S1656	ROX^™^	F: GTGTTGCTCAAGGGTCAACTGTATG	0.2	62.8	115–170
		R: AGAGAAATAGAATCACTAGGGAACC	0.2	57.1	
D7S3048	ROX^™^	F: GCATTGCCACTTCCCCCTG	0.5	63.1	175–233
		R: CCCCCGCAGTCAAAAATCT	0.5	59.8	
D10S1248	ROX^™^	F: GAGCATTAGCCCCAGGACCA	0.5	62.2	234–280
		R: TGCAGTGCTTGGCAAAGAGC	0.5	62.2	
D19S253	ROX^™^	F: CTCCATGAACAGACAGTTTGTCTTC	0.5	59.9	355–420
		R: CTTTTCAGCTTCCATAAACATGAGT	0.5	59.4	
