# Supplementary material for: A New Multiplex Assay of 17 Autosomal STRs and Amelogenin for Forensic Application
Source: PLoS One. 2013 Feb 25;8(2):e57471. doi: 10.1371/journal.pone.0057471 (PMC3581461; doi:10.1371/journal.pone.0057471)
Supplement: Table S1 — General information for the 17 autosomal STRs included in the final multiplex. (DOCX) [file pone.0057471.s001.docx]

Table S1 General information for the 17 autosomal STRs included in the final multiplex

| Locus | UniSTS id | GenBank accession number | Chromosomal mapping | Dye | Genotyping results of 9947A |
| --- | --- | --- | --- | --- | --- |
| D6S477 | 46470 | G08543 | 6p24.6 | FAM | 10.2，13 |
| D22-GATA198B05 | 498563 | [AC135612.26](http://www.ncbi.nlm.nih.gov/nucleotide/84662974) | 22q11.15 | FAM | 18，19 |
| D15S659 | 58271 | G07907 | 15q20 | FAM | 14，16 |
| D8S1132 | 53216 | G08685 | 8q22.6 | FAM | 19，21 |
| D3S1358 | 148226 | GDB:196594 | 3p21.31 | HEX | 14，15 |
| D3S3045 | 8620 | G08279 | 3q13.1 | HEX | 9，9 |
| D17S1290 | 48243 | G07956 | 17q23 | HEX | 17，18 |
| D14S608 | 60255 | G09052 | 14q12 | HEX | 7，11 |
| D2S441 | 71306 | G08184 | 2p14.5 | TEM | 10，14 |
| D18S535 | 65584 | G07985 | 18q12.5 | TEM | 13，14 |
| D13S325 | 67237 | G09015 | 13q13.7 | TEM | 20，21 |
| D10S1435 | 61895 | G08819 | 10p15.3 | TEM | 10，11 |
| D11S2368 | 50 | G08890 | 11p14.5 | TEM | 19，21 |
| D1S1656 | 58809 | G07820 | 1q42.3 | ROX | 18.3，18.3 |
| D7S3048 | 38746 | G10359 | 7p17.5 | ROX | 24，24 |
| D10S1248 | 51457 | G08820 | 10q26.5 | ROX | 13，15 |
| D19S253 | 147765 | GDB:190893 | 19p13.1 | ROX | 7，8 |
